# The risk factors and care measures of surgical site infection after cesarean section in China: a retrospective analysis

**DOI:** 10.1186/s12893-021-01154-x

**Published:** 2021-05-19

**Authors:** Lijun Li, Hongyan Cui

**Affiliations:** Department of Obstetrics, Tianjin Central Hospital of Gynecology Obstetrics, No. 156 Nankai three Road, Nankai District, Tianjin, China

**Keywords:** Pathogen, Incision, Infection, Cesarean section, Nursing

## Abstract

**Background:**

Surgical site infections after cesarean section are very common clinically, it is necessary to evaluate the risk factors of surgical site infections after cesarean section, to provide evidences for the treatment and nursing care of cesarean section.

**Methods:**

This study was a retrospective cohort study design. Patients undergone cesarean section in a tertiary hospital of China from May 2017 to May 2020 were identified, we collected the clinical data of the included patients, and we analyzed the infection rate, etiological characteristics and related risk factors of surgical site infection after caesarean section.

**Results:**

A total of 206 patients with cesarean section were included, and the incidence of surgical site infection in patients with cesarean section was 23.30%. A total of 62 cases of pathogens were identified, *Enterococcus faecalis* (33.87%) and *Escherichia coli* (29.03%) were the most common pathogens. Both *Enterococcus faecalis* and *Escherichia coli* were highly sensitive to Cefoperazone, Meropenem, and Levofloxacin. Logistic regression analyses indicated that Age ≥ 30y (OR 4.18, 95%CI: 1.23–7.09), BMI ≥ 24 (OR 2.39, 95%CI: 1.02–4.55), duration of cesarean section ≥ 1.5 h (OR 3.90, 95%CI: 1.28–5.42), estimated blood loss ≥ 400 ml (OR 2.35, 95%CI: 1.10–4.37) and the duration of urinary catheter ≥ 24 h (OR 3.18, 95% CI: 1.21–5.71) were the independent risk factors of surgical site infection after cesarean section (all p < 0.05).

**Conclusions:**

Age, BMI, duration of surgery, blood loss and urinary catheter use were associated with higher risk of the surgical site infection after cesarean section. Clinical preventions and interventions are warranted for those population to reduce the occurrence of surgical site infection.

## Background

With advances of blood transfusion, anesthesiology, diversification of surgical methods, improvement of surgical suture materials, cesarean section has become an effective treatment of maternal women [ [1, 2]]. However, the risks of postoperative thrombosis, intraoperative bleeding, uterine rupture, and placenta previa of re-pregnancy after cesarean delivery are significantly higher than those of women delivered via vaginal delivery [3–5]. Besides, the surgical site infection after cesarean section is a common yet serious complication, which has raised the attentions of health care providers [6]. It’s been reported the incidence of surgical site infection after cesarean section is relatively high, which ranges from 8.18 [7] to 39.17% [8]. Surgical site infection not only affect the prognosis of pregnant women, but also may involve abdominal organs, and even induce diffuse abdominal infections, thereby prolonging the length of hospitalization, and bringing greater psychological and economic burden to the mothers and their families [9–11]. Therefore, it’s essential to promptly detect the risk factors of surgical site infection after cesarean section, and to understand the etiological characteristics of surgical site infection after cesarean section. Therefore, we aimed to assess the pathogenic characteristics and risk factors of surgical site infection after cesarean section, to provide insights into the management of patients with cesarean section.

## Methods

### Ethical issue

This study passed the ethical review of the medical ethics committee of Tianjin central hospital of gynecology obstetrics (170,093), and all patients signed the written informed consents.

### Participants

Patients undergone cesarean section in our hospital from May 2017 to May 2020 were considered as potential participants, our study is a tertiary hospital specialized in gynecology and obstetrics in Tianjin, China. The inclusion criteria were: ①adult patients with age ≥ 22y; ②the patients underwent cesarean section in our hospital, and the medical records were kept intact; ③patient agreed to participant in our study. The exclusion criteria were: ① patients with severe heart, brain, liver, kidney and other serious disorders; ② patients with tuberculosis or malignant tumor disease; ③patients with local infection or systemic infection symptoms before the admission; ④patients who received the antibacterial treatment before the culture of secretions; ⑤patients who didn’t agree to participant in this study.

### Diagnosis of surgical site infection

The diagnostic criteria for surgical site infection were complied with related guidelines [12, 13]: ①the patient's body temperature was higher than 38℃; ②the percentage of neutrophils was higher than 70%, and the white blood cell count was less than 4. 0 × 10^9^/L or higher than 10.0 × 10^9^/L; ③the pathogenic bacteria were cultivated in the incision secretions.

### Pathogen detection

We cleaned the incision site with sterile saline, and disposable sterile cotton swab was used to collect 2 mL of incision secretion, and placed it in a sterile test tube after successful collection, and immediately sent it for inspection. Four nurses collected the swab, we conducted a strict training on the specimen collection to make sure the consistency of specimen collection. We intensively cultivated specimens in a suitable medium and cultivate overnight at 35 °C. The BIOFoS automatic bacterial identification instrument (Meisher Diagnostics Co., Ltd., Shanghai) was used to analyze the distribution of pathogens [14]. The pathogenic cultivation, identification and drug sensitivity analysis of the pathogens were conducted in accordance with the relevant procedures [15–17], we had taken 25% as the resistance threshold [18]. When the culture result is positive, we isolated and purified the bacteria to analyze and detect the protein components of the strains. After obtaining the characteristic peak spectrum, we collected the peak spectrum and record, and compared it with the pre-stored bacterial profiles in the detection system (Henrui DS2000, Shanghai, China) and made a positive diagnosis based on the degree of agreement between pre-stored bacterial and detected spectrum [19].

### SSI preventions

Our hospital strictly controlled the antibiotics use, and all the included patients did not accept the pre-operative antibiotic prophylaxis. We developed health education materials for medical staff and patients' families. Through regular lectures (once every 3 months) to improve the skills of medical staff in infection risk assessment, early identification and control. Besides, nurses were required to talk face-to-face with the patients and their families about the hazards of infection, prevention methods and related precautions, and they distributed the materials of infection prevention. In addition, for cases of surgical site infection, relevant doctors and nurses were required to review the surgical process and analyze patient-related information to discuss the possible causes of its occurrence, and proposed related control recommendations. On this basis, special seminars were organized to discuss correct hand washing methods, disinfection and sterilization techniques, and standardize the skin preparation process. Additionally, we informed the patient that the skin around the incision should be kept clean.

### Data collection

We made a follow-up in the period of patients’ stay in hospital, and we collected many personal data and treatment data of patients from the admission record and treatment plans, including age, body mass index (BMI), intraoperative blood loss, duration of cesarean section, estimated blood loss and the duration of urinary catheter, the pathogens of surgical site infection and resistant rate of bacteria to antibiotics.

### Data analysis

All of the data analyses were conducted with SPSS 22.0 (SPSS Inc., Chicago, USA). Categorical variables were analyzed with χ^2^ test or Fisher’s exact test, and continuous variables were analyzed with Student’s t test. p < 0.1 was the significance level to select variables in this study. Multivariate logistic regression analyses were performed using the forward likelihood ratio selection method to identify independent risk factors. Besides, p < 0.05 was considered as being statistically significant in this study.

## Results

### The characteristics of included patients

A total of 206 patients with cesarean section were included. And 48 patients were diagnosed with surgical site infection, the incidence of surgical site infection was 23.30%. The characteristics of included patients were presented in Table 1.

**Table 1:** The characteristics of included patients (n = 206)

Items	
Age (y)	31.24 ± 6.01
BMI (kg/m^2^)	24.11 ± 3.84
Mean gestational age (w)	35.94 ± 4.17
Duration of cesarean section (h)	1.25 ± 0.74
Estimated blood loss (ml)	387.34 ± 40.89
The duration of urinary catheter (h)	22.82 ± 4.94

### Pathogens distribution

As Table 2 presented, a total of 62 cases of pathogens were identified, *Enterococcus faecalis* (33.87%) and *Escherichia coli* (29.03%) were the two main pathogens.

**Table 2:** Distribution of pathogens of surgical site infection after cesarean section (n = 62)

Pathogens	Number	Proportion (%)
Gram-positive bacteria	32	51.61
*Enterococcus faecalis*	21	33.87
Staphylococcus aureus	5	8.06
Hemolytic Streptococcus	4	6.45
Staphylococcus epidermidis	2	3.23
Gram-negative bacteria	27	43.55
*Escherichia coli*	18	29.03
Pseudomonas aeruginosa	5	8.06
Enterobacter cloacae	2	3.23
Clostridium perfringens	2	3.23
Fungus	3	4.84
Candida albicans	3	4.84

*Enterococcus faecalis* were highly sensitive to Cefoperazone, Meropenem, and Levofloxacin. And *Enterococcus faecalis* were highly resistant to Gentamicin, Ampicillin, Sulfamethoxazole, Amoxicillin and Ceftriaxone

### The resistant rate and antibiotic sensitivity

As Table 3 presented, *Enterococcus faecalis* were highly sensitive to Cefoperazone, Meropenem, and Levofloxacin. And *Enterococcus faecalis* were highly resistant to Gentamicin, Ampicillin, Sulfamethoxazole, Amoxicillin and Ceftriaxone. *Escherichia coli* were highly sensitive to Cefoperazone, Meropenem, and Levofloxacin, Cefoxitin and Norfloxacin. And *Escherichia coli* were highly resistant to Sulfamethoxazole, Ampicillin, Amoxicillin and Ceftriaxone.

**Table 3:** The resistant rate and antibiotic sensitivity of *Enterococcus faecalis* and *Escherichia coli* to antibiotics

Antibiotics	*Enterococcus faecalis* and *Escherichia coli* to antibiotics (n = 21)			*Escherichia coli* to antibiotics (n = 18)		
	Cases	Resistant rate (%)	Antibiotic sensitivity (%)	Cases	Resistant rate (%)	Antibiotic sensitivity (%)
Levofloxacin	2	7.48	89.14	1	2.13	90.15
Gentamicin	17	73.22	13.87	8	39.55	52.09
Cefepime	5	20.18	77.12	3	10.14	78.33
Piracetam	5	21.94	70.25	3	9.85	87.06
Cefoperazone	0	0	91.18	0	0	93.78
Ampicillin	16	78.03	20.11	11	53.12	33.31
Meropenem	0	0	91.74	0	0	90.27
Sulfamethoxazole	17	74.17	22.08	14	67.09	27.44
Cefoxitin	2	8.72	81.26	1	4.95	86.42
Amoxicillin	13	68.90	30.13	16	74.27	11.29
Ceftriaxone	13	69.17	23.95	11	58.39	40.77
Norfloxacin	2	9.03	87.14	1	4.86	84.02

### Single-variate analysis

As Table 4 presented, there were significant differences in the Age ≥ 30y, BMI ≥ 24, duration of cesarean section ≥ 1.5 h, estimated blood loss ≥ 400 ml and the duration of urinary catheter ≥ 24 h between two groups (all p < 0.05), and no significant difference on the mean gestational age ≤ 35 w were found (all p = 0.091).

**Table 4:** Single variate analysis on the risk factors of surgical site infection after cesarean section

Items	Infection group (n = 48)	No-infection group (n = 158)	χ^2^	p
Age ≥ 30y	39 (81.25%)	82 (51.89%)	1.128	0.033
BMI ≥ 24	34 (70.83%)	67 (42.41%)	1.096	0.029
Mean gestational age ≤ 35 w	15 (31.25%)	48 (30.38%)	1.631	0.091
Duration of cesarean section ≥ 1.5 h	23 (47.92%)	41 (25.95%)	1.107	0.017
Estimated blood loss ≥ 400 ml	26 (54.17%)	19 (12.06%)	1.266	0.024
The duration of urinary catheter ≥ 24 h	22 (45.83%)	34 (21.52%)	1.182	0.038

### Logistic regression analysis

As Table 5 presented, the logistic regression analyses indicated that Age ≥ 30y (OR4.18, 95%CI: 1.23–7.09), BMI ≥ 24 (OR2.39, 95%CI: 1.02–4.55), duration of cesarean section ≥ 1.5 h (OR3.90, 95%CI: 1.28–5.42), estimated blood loss ≥ 400 ml (OR2.35, 95%CI: 1.10–4.37) and the duration of urinary catheter ≥ 24 h (OR3.18, 95%CI: 1.21–5.71) were independent risk factors of surgical site infection after cesarean section (all p < 0.05).

**Table 5:** The results of logistic regression analysis

Factors	β	S‾x	OR	95%CI	p
Age ≥ 30y	0.85	0.23	4.18	1.23–7.09	0.018
BMI ≥ 24	0.99	0.13	2.39	1.02–4.55	0.027
Duration of cesarean section ≥ 1.5 h	0.83	0.23	3.90	1.28–5.42	0.009
Estimated blood loss ≥ 400 ml	0.66	0.17	2.35	1.10–4.37	0.042
The duration of urinary catheter ≥ 24 h	0.79	0.20	3.18	1.21–5.71	0.012

## Discussion

Surgical site infection is one of the common complications of cesarean section, which not only can affect the rehabilitation process, but also extend the length of hospital stay and increase medical-related expenses [20]. Previous studies [21, 22] have reported that the incidence of nosocomial infection in pregnant women after cesarean section is higher than that of women with transvaginal delivery, and the rate of surgical site infections after cesarean section can be as high as 39.86%. In this study, it was found that 23.30% postoperative surgical site infections occurred in pregnant women with cesarean section, which indicates that the risk of surgical site infections after cesarean section is relatively high. It is worth paying attention to the etiological characteristics and related risk factors of postpartum surgical site infection, which are crucial for early prevention and treatment of surgical site infection [23]. The results of this study have indicated that the age ≥ 30y, BMI ≥ 24, duration of cesarean section ≥ 1.5 h, estimated blood loss ≥ 400 ml and the duration of urinary catheter ≥ 24 h were the risk factors for those patients, early preventative strategies are needed for the prophylaxis of surgical site infection.

The etiological characteristics of surgical site infection after cesarean section indicate that Gram-negative bacteria account for 51.61%, Gram-positive bacteria account for 43.55%, and fungi account for 4.84%. And in the related drug sensitivity test, both *Enterococcus faecalis* and *Escherichia coli* were highly sensitive to Cefoperazone, Meropenem, and Levofloxacin. The etiological characteristics of surgical site infection can provide clinical guidance for medication and help to better control of infection [24, 25]. Therefore, early bacterial culture of infected wounds and rational use of antibacterial drugs are of great significance for reducing bacterial resistance and extending the window of antibacterial drugs use.

The several risk factors related to surgical site infections should be concerned. Due to the continuous liberalization of China’s second child policy, the maternal age is uneven, and the women with older age have relatively poor tolerance to surgery and immunity, which is an important risk factor for surgical site infection after cesarean section [26]. Besides, during the surgery, a large amount of bleeding may occur, which may cause anemia and even hemorrhagic shock [27]. Blood is an ideal medium for bacterial reproduction, which will increase risk of infection and delay the incision healing [28]. Women with higher BMI tend to have relatively less activity, and long-term bed rest after surgery will affect the body's blood circulation, thereby increasing the risk of surgical site infection [29]. Furthermore, women's urinary tract is closely adjacent to the reproductive tract, but when a urinary tract infection occurs, it easily spreads to the reproductive system, thereby increasing the probability of infection [30]. The longer the urinary catheter stays, the greater the risk of surgical site infection [31].

There are internal and external factors that affect the surgical site infection after cesarean section, and many factors are controllable [32]. The following effective preventive measures may be beneficial. Strengthening health education during pregnancy and conducting regular prenatal examinations to understand the conditions of pregnant women [33]. Obese women should initially control the diet, strengthen exercise to balance the BMI [34–36]. Before surgery, patients should be encouraged to urinate on their own [37]. Those who have serious urination problems can be pre-operatively catheterized, but the urinary catheter should be removed as soon as possible [38]. Related studies [39, 40] have shown that the probability of urinary tract infection is as high as more than 90% if the catheterization time exceeds 3 days. Women should be encouraged to choose natural vaginal births whenever possible [41].

## Conclusions

In conclusion, for women with age ≥ 30y, BMI ≥ 24, duration of cesarean section ≥ 1.5 h, estimated blood loss ≥ 400 ml and the duration of urinary catheter ≥ 24 h during the cesarean section, targeted intervention should be performed to prevent the surgical site infection. Controlling BMI, reduce the duration of cesarean section, blood loss, duration of urinary catheter is beneficial to the prevention of surgical site infections. However, we do have several limitations in this present study. We have excluded those patients because patients with co-morbidities may have higher risks of surgical site infection in many aspects, it may lead to selection biases, the results should be treated with cautions. Besides, limited by small sample size, more studies are warranted to further elucidate the risk factors of surgical site infection after cesarean section, to provide evidences for the management of cesarean section.

## Data Availability

All data generated or analysed during this study are included in this published article and its supplementary information files.

## Abbreviation

BMI: Body mass index
